# Milking It for All It’s Worth: The Effects of Environmental Enrichment on Maternal Nurturance, Lactation Quality, and Offspring Social Behavior

**DOI:** 10.1523/ENEURO.0148-22.2022

**Published:** 2022-08-25

**Authors:** Holly DeRosa, Salvatore G. Caradonna, Hieu Tran, Jordan Marrocco, Amanda C. Kentner

**Affiliations:** 1 School of Arts & Sciences, Health Psychology Program, Massachusetts College of Pharmacy and Health Sciences, Boston, MA 02115; 2 Laboratory of Neuroendocrinology, The Rockefeller University, New York, NY 10065; 3 Department of Biology, Touro University, New York, NY 10023

**Keywords:** environmental enrichment, maternal brain, microbiome, milk quality, postnatal experience, RNA-sequencing

## Abstract

Breastfeeding confers robust benefits to offspring development in terms of growth, immunity, and neurophysiology. Similarly, improving environmental complexity, i.e., environmental enrichment (EE), contributes developmental advantages to both humans and laboratory animal models. However, the impact of environmental context on maternal care and milk quality has not been thoroughly evaluated, nor are the biological underpinnings of EE on offspring development understood. Here, Sprague Dawley rats were housed and bred in either EE or standard-housed (SD) conditions. EE dams gave birth to a larger number of pups, and litters were standardized and cross-fostered across groups on postnatal day (P)1. Maternal milk samples were then collected on P1 (transitional milk phase) and P10 (mature milk phase) for analysis. While EE dams spent less time nursing, postnatal enrichment exposure was associated with heavier offspring bodyweights. Milk from EE mothers had increased triglyceride levels, a greater microbiome diversity, and a significantly higher abundance of bacterial families related to bodyweight and energy metabolism. These differences reflected comparable transcriptomic changes at the genome-wide level. In addition to changes in lactational quality, we observed elevated levels of cannabinoid receptor 1 in the hypothalamus of EE dams, and sex-dependent and time-dependent effects of EE on offspring social behavior. Together, these results underscore the multidimensional impact of the combined neonatal and maternal environments on offspring development and maternal health. Moreover, they highlight potential deficiencies in the use of “gold standard” laboratory housing in the attempt to design translationally relevant animal models in biomedical research.

## Significance Statement

Maternal care quality is different between environmental enrichment (EE) and standard laboratory housed (SD) dams. SD rat dams spend more time nursing their young. This may result in metabolic differences in milk quality and affect the neurodevelopmental outcomes of offspring, which are different between EE and SD animals. To test this, we evaluated milk and offspring behavior. Milk from EE dams had elevated triglyceride levels and microbiome diversity. EE offspring had heavier body weights and increased social behavior which was lost with cross-fostering into SD housing. These data identify potential deficiencies in the quality of “gold standard” laboratory housing and its impact on the welfare and design of translationally relevant animal models in biomedical research.

## Introduction

The maternal-infant interaction of breastfeeding is a critical component in the neurodevelopment of offspring. Breastmilk promotes increased white and gray matter volume, improved cortical thickness (Isaacs et al., 2010; Luby et al., 2016) and neuromuscular development (Grace et al., 2017). Milk may facilitate development of the central nervous system by influencing genes related to neural growth and maturation (Huff et al., 2020). Additionally, milk microbes can prime infant intestinal tract and gut microbiome development (Sheard and Walker, 1988; Pannaraj et al., 2017). While this evidence implicates milk in physiological development, its role in offspring behavior needs further elucidation.

Breastmilk composition can be modulated by the maternal environment, and several studies revealed the pervasive effects of stress on milk production and quality. For example, mothers exposed to natural disasters often report a reduction or sometimes complete loss of lactation (Adhisivam et al., 2006; DeYoung et al., 2018). Psychosocial stress is also negatively associated with maternal milk fat and energy content (Ziomkiewicz et al., 2021) and mothers who reported higher levels of perceived stress exhibited significantly lower levels of milk immunoglobulin (Ig)A (Groer et al., 2004; Moirasgenti et al., 2019). Likewise, environmental stressors correlate with altered maternal milk quality in laboratory animals. Restraint stress reduced milk protein in lactating mice (Chiba et al., 2019), and other stressors such as social and heat stress negatively affected lactation (Murgatroyd et al., 2015) and milk yield (Haldar and Bade, 1981). Such evidence underscores how the maternal environment may alter the nutritional profile of milk which may have subsequent consequences on progeny development.

The enhancement of environmental complexity, i.e., environmental enrichment (EE), is employed in human populations to promote cognitive plasticity (Baroncelli et al., 2010; Kentner et al., 2019a,b; Tooley et al., 2021). Additionally, EE housing reduces stress and stereotypy and promotes species typical behaviors in the animal laboratory. This enhanced housing condition affects the display of rodent maternal care behaviors (Cancedda et al., 2004; Sale et al., 2004; Welberg et al., 2006; Mann and Gervais, 2011; Connors et al., 2015; Strzelewicz et al., 2019) which are central to the development of effective stress regulation and health of the offspring (Meaney and Szyf, 2005; Meaney, 2010; Buschdorf and Meaney, 2015). Research supports the notion that EE dams are more efficient mothers compared with their standard-housed (SD) counterparts. Indeed, while rats housed in EE spent less time on their nest compared with SD dams, both groups licked and groomed their pups at a similar (Welberg et al., 2006; Connors et al., 2015; Strzelewicz et al., 2019) or even higher frequency (Sale et al., 2004), although these findings are not always consistent (Cancedda et al., 2004; Rosenfeld and Weller, 2012). Additionally, EE dams spend less time in passive nursing postures compared with SD dams and demonstrate higher levels of the more effective active, or high arched back nursing posture (Connors et al., 2015; Strzelewicz et al., 2019). In another study, rat dams housed in cages with a loft that afforded an opportunity to periodically leave their pups also exhibited lower levels of passive nursing (Ratuski and Weary, 2021). This suggests that time away from the pups promotes an efficient maternal care style on returning to the nest. This is congruent with what is typically observed under naturalistic conditions, where wild rat dams will leave their nests for extended periods to forage and defend their territory (Grota and Ader, 1969; Hughes et al., 1978).

Given that SD dams housed in the classic “gold standard” laboratory housing condition spend more time on the nest suggests they may overfeed their offspring. Alternatively, there may be metabolic differences in milk quality where SD offspring require more nourishment, necessitating longer nursing periods. Indeed, dams in cages with reduced opportunities to leave their litters spend more time in a “press posture” position with the ventral surface of their body pressed against the cage, hiding their teats from their pups (Cramer et al., 1990; Gaskill and Pritchett-Corning, 2015). These considerations have important implications for laboratory animal health and stress regulation. Since early life manipulations as modest as experimenter handling exert transgenerational alterations in rodent parenting behavior (Stone and Bales, 2010), understanding how environmental manipulations impact parental input signals, like maternal care and breastfeeding, is imperative to the translational relevance of our animal models. Therefore, in the current study we explored the effects of EE versus SD housing on rodent maternal care, maternal milk quality, and offspring body weight and social behavior outcomes in male and female rats.

## Materials and Methods

### Animals and housing

**Figure 1.**
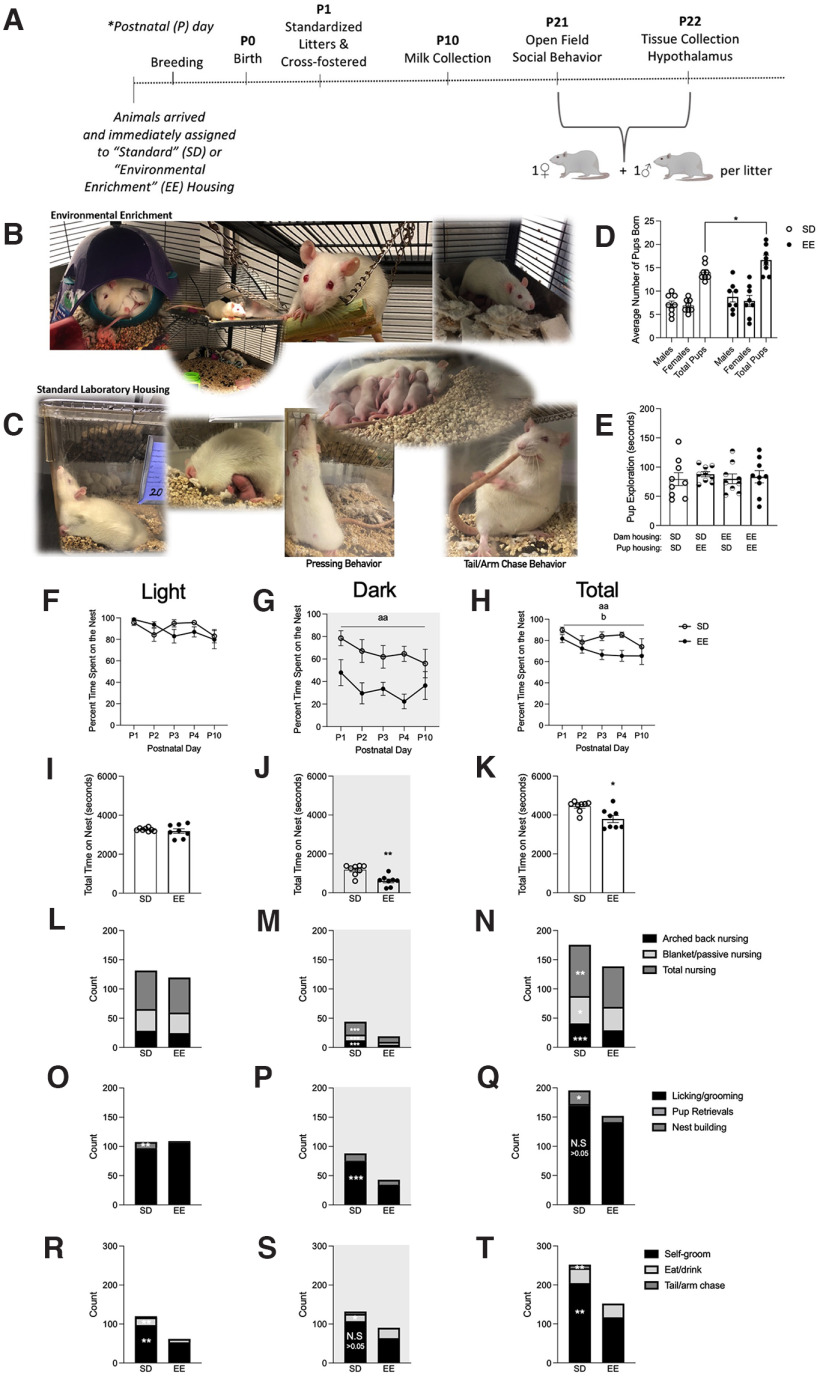
Maternal care behaviors are different between EE and SD Sprague Dawley rat dams. ***A***, Timeline of experimental procedures. ***B***, Representative photographs of EE housing and litters. ***C***, Representative photographs of SD housing and litters. ***D***, Average number of pups born (male, female, and total pups) per SD and EE housing group (*n* = 8). ***E***, Total time (seconds) SD and EE housed dams spent exploring P7 alien pups from different housing conditions (*n* = 9). Percent of time that dams spent on the nest across P1–P4 and P10 in the (***F***) light, (***G***) dark, and (***H***) light + dark periods combined. Total time (seconds) that dams spent on the nest collapsed across P1–P4 and P10 in the (***I***) light, (***J***) dark, and (***K***) light + dark periods combined. Stacked bars depict the frequency of pup directed nursing behaviors (arched back nursing, blanked/passive nursing, total nursing) collapsed across P1–P4 and P10 in the (***L***) light, (***M***) dark, and (***N***) light + dark periods. Stacked bars depict the frequency of other types of pup directed behaviors (licking/grooming, pup retrievals, nest building behaviors) collapsed across P1–P4 and P10 in the (***O***) light, (***P***) dark, and (***Q***) light + dark periods. Stacked bars depict the frequency of maternal self-directed behaviors (self-grooming, eating/drinking, tail/arm chases) collapsed across P1–P4 and P10 in the (***R***) light, (***S***) dark, and (***T***) light + dark periods (*n* = 8). Data are expressed as mean ± SEM; SD: open circles versus EE: closed circles. *p < 0.05, **p < 0.01, ***p < 0.001, SD versus EE; ^aa^*p* < 0.01, main effect of housing; ^b^*p* < 0.05, main effect of postnatal day. See Extended Data Figure 1-1.

Sprague Dawley rats (Charles River, Wilmington, MA) were maintained at 20°C on a 12/12 h light/dark cycle (7 A.M. to 7 P.M.) with *ad libitum* access to food and water. Figure 1*A* outlines the experimental procedures followed in this study. Female animals were pair-housed in one of two conditions: EE (91.5 × 64 × 159 cm; Fig. 1*B*), which was a large multi- level cage with ramps and access to toys, tubes, chew bones, and Nestlets (Ancare), or standard laboratory cages (SD; 27 × 48 × 20 cm; Fig. 1*C*). Three enrichment toys (e.g., small plastic balls, climbing ropes and ladders, swings, bell rollers, chew toys, hammocks, additional tubes/tunnels, Lixit Space Pods, cups, and other small animal hideaways) were switched out twice weekly to maintain novelty in the EE condition.

10.1523/ENEURO.0148-22.2022.f1-1 Extended Data Figure 1-1 Maternal factors in rats housed in standard laboratory housing (SD) or EE environments. Plasma concentrations of prolactin (ng/ml; ***A***), P10 body weights (***B***), and total number of eating bouts (***C***) from P1 to P4 and P10 during the light and dark phases for dams housed in EE or SD housing. Data are expressed as mean ± SEM; *p < 0.05, main effect of housing (SD vs EE), *n* = 7–8. SD (open circles) or EE (closed circles). Download Figure 1-1, TIF file.

Male rats were paired in SD conditions unless they were breeding, which occurred two weeks after animals were introduced to their housing condition. During breeding, they were housed with two females in either EE housing or in larger SD one-level cages (51 × 41 × 22 cm) with access to a tube, one chew bone and Nestlets (Ancare). Approximately 2 d before parturition, dams in the SD condition were individually housed (27 × 48 × 20 cm; Fig. 1*C*), while a physical divider separated the EE dams within their cage (allowing for auditory, tactile, olfactory, and some visual contact; important components of EE). This separation prevented the mixing of litters. Day of birth was designated as postnatal day (P)0 and litters were standardized to 10 pups (five males and five females) per litter on P1. To dissociate the effects of the prenatal and postnatal housing environments, male and female pups from each litter were cross-fostered, replacing an age-matched and sex-matched pup in the litter they were fostered to. To track the housing of origin, pups were marked on their left or right ear to indicate prenatal SD or EE respectively, resulting in the following study group designations: SD-SD, SD-EE, EE-SD, EE-EE. Offspring were maintained in these respective housing conditions until the end of the study on P22. The MCPHS University Institutional Care and Use Committee approved all procedures described, which were conducted in compliance with the recommendations outlined by the *Guide for the Care and Use of Laboratory Animals* of the National Institutes of Health.

### Maternal care

Maternal behavior observations took place between P1–P4 and again on P10 following the milking procedures (*n* = 8). Sessions occurred three times daily (7:30 A.M., 3 P.M., 8 P.M.), consisting of six observations that were summed together to create a composite score for each dam and observation period for each behavior evaluated. Dams were evaluated for 1 min intervals per observation, with at least 5 min of no observations occurring between each of the 1 min bins. Maternal care observations recorded included the frequency of pup-directed behaviors (i.e., dam licking/grooming pups, active/high crouch nursing, passive/low crouch nursing, pup retrieval), self-directed behaviors (i.e., dam eating/drinking, dam self-grooming, dam chasing her arm/tail), and nest building/digging behavior. Total time the dam spent on her nest (seconds) was also recorded (Connors et al., 2015; Strzelewicz et al., 2019, 2021).

### Milk sample collection

To encourage the accumulation of maternal milk for sample collection, on the mornings of P1 (equivalent to a transitional milk phase) and P10 (mature milk phase), dams were removed from their litter and placed into a clean cage in a separate procedure room for 1 h. Pups remained in their regular holding room and were placed into a smaller clean cage positioned on top of a heating pad, to maintain their body temperature. Litters were weighed immediately before being returned to their dams and again 2 and 24 h later, alongside the inspection of milk bands, to monitor their health.

The milk procedure was adapted from a published procedure (Paul et al., 2015). Immediately following the separation period, dams were lightly anesthetized with isoflurane in O_2_, followed by the administration of 0.2 ml of oxytocin (20 USP/ml, i.p.). Distilled water was used to moisten the teats and milk obtained by gently squeezing its base to manually expel the sample for collection. A microhematocrit tube was used to collect ∼20 μl of sample. The tube was then sealed and placed into a hematocrit spinner and spun for 120 s at 13,700 × *g*. Measurements of the separation of the milk into cream and clear layers were taken to calculate percent (%) creamatocrit using the procedures outlined in Paul et al. (2015). The remaining milk collected (∼500 μl per animal) was transferred to small centrifuge tubes and stored at −80°C until processing. Collection time took ∼10–20 min per animal, and dams were returned to their litter as soon as they awoke from anesthesia. This was appropriate as breastfeeding can resume immediately after isoflurane anesthesia since the pharmacokinetics of the compound indicate it is poorly absorbed by infants (Lee and Rubin, 1993; Drugs and Lactation Database, 2006). With respect to oxytocin, its typical half-life (1–6 min) is reduced even further during lactation and this drug is also unlikely to affect offspring (Par Pharmaceutical, Inc., 2020).

### Milk sample immunoassays

Milk samples (*n* = 8) were placed onto a Mini Tube Rotator (Fisher Scientific catalog #88861051) overnight at 4°C to homogenize before analysis. Following the standard manufacturer’s instructions, commercially available ELISA kits were used to measure lactose content (Sigma-Aldrich catalog #MAK017; diluted 1:500; as outlined by Y. Chen et al., 2017; DeRosa et al., 2022), triglycerides (Abcam, catalog #ab65336; 1:1000 dilution), protein (Pierce BCA Protein Assay kit; catalog #23227; 1:50 dilution), and IgA (Bethyl Laboratories, catalog #E111-102; diluted to 1:1000) levels in milk. To measure corticosterone, the small sample assay protocol (#ADI-900-097, Enzo Life Sciences) was followed, as recommended by the manufacturer, using a 1:40 dilution (DeRosa et al., 2022). We opted to only evaluate P10 milk samples on these measures because litters were appropriately standardized in size for this time point.

### Microbiome sequencing of milk samples

Another subset of P10 milk samples underwent microbiome community analysis (*n* = 6). Milk DNA was first extracted using the ZymoBIOMICS −96 MagBead DNA kit (Zymo Research). The *Quick*-16S NGS Library Prep kit (Zymo Research) was used for bacterial 16S ribosomal RNA-targeted sequencing and custom 16S primers were used to amplify the V3–V4 region (Zymo Research). Real-time PCR was then used to prepare the sequencing library and final qPCR fluorescence readings were pooled together according to equal molarity and the final pooled library was cleaned using the Select-a-Size DNA Clean & Concentrator (Zymo Research), and quantified with TapeStation (Agilent Technologies) and Qubit (ThermoFisher Scientific). Illumina MiSeq with a v3 reagent kit (600 cycles) was used along with a 10% PhiX spike-in to sequence the final library. Unique amplicon sequences, as well as possible sequencing errors and chimeric sequences, were inferred from raw reads using the DADA2 pipeline (Callahan et al., 2016). Uclust from Qiime (v.1.9.1) was used to determine taxonomy assignment and referenced with the Zymo Research Database (Zymo Research).

### RNA-sequencing (RNA-seq) of milk samples

The fat layer of breastmilk is known to contain epithelial RNA (Lemay et al., 2013; Alsaweed et al., 2015). To investigate maternal milk at the genome-wide level, total RNA (*n* = 4–5 per housing group) was isolated from the milk fat layer from a subset of P10 milk samples using a previously published procedure (Y. Chen et al., 2017; DeRosa et al., 2022). Milk was centrifuged for 5 min at 1000 × *g* (4°C) and the fat layer isolated. Milk fat was then mixed with an equal volume of PBS by centrifuging at 3000 rpm (5 min in 4°C), cleaning the fat of debris. The miRNeasy Mini kit (QIAGEN, catalog #217004) was used to isolate total RNA following the manufacturer’s directions. A NanoDrop 2000 spectrophotometer (ThermoFisher Scientific) was used to quantify isolated RNA samples, which were then stored at −80°C. For RNA-seq, the quality of the RNA was determined using Bioanalyzer (Agilent Technologies). The cDNA library was compiled using the TruSeq Stranded mRNA kit (Illumina), and processed through Tapestation to determine fragment size and DNA concentration. The library was then sequenced on an Illumina NovaSeq 6000 to obtain single-end 100-bp reads. Samples were read at a sequencing depth of ∼50 million reads. These reads were then aligned to the rn6 genome using the BSgenome.Rnorvegicus.UCSC.rn6 R/Bioconductor package (version 1.4.1). Differentially expressed genes, using a *p* < 0.05, Benjamini–Hochberg false discovery rate (FDR) corrected, and fold change (FC) > 1.3, were identified using DESeq2 package (Love et al., 2016). Heatmaps were generated using the *z* score of rlog-normalized counts and were plotted with the MultiExperiment Viewer (National Library of Medicine). Gene ontology from genes with *p* < 0.05 or FDR adjusted *p* < 0.05 with a FC > 1.3 was generated using the Database for Annotation, Visualization and Integrated Discovery functional annotation cluster tool (https://david.ncifcrf.gov/). RNA-seq data have been deposited to GEO (GSE200249).

### Open field and social preference tests

During the light phase on P21, one male and one female offspring per litter from each prenatal versus postnatal housing condition was habituated to an open field arena for 3 min (40 × 40 × 28 cm; Duque-Wilckens et al., 2020; Williams et al., 2020; *n* = 7–8). Behavior was recorded and videos scored using an automated behavioral monitoring software program (Cleversys TopScan) to determine total distance traveled (cm) and percent of time spent in the center of the arena. All equipment was thoroughly cleaned with Quatriside TB between each animal and test. Immediately after the open field habituation period, animals were evaluated in a 5-min social preference test. Using the manual behavioral monitoring program ODLog 2.0 (https://www.macropodsoftware.com/odlog/), animals were evaluated on their choice to visit a novel inanimate object or a novel same sex and age conspecific, each enclosed within a small wire cup on opposite ends of the arena (Crawley, 2007). Placement of novel rats and objects were interchanged between trials and experimental groups counterbalanced between tests. Animals were recorded as actively investigating when their nose was directed within 2 cm of a containment cup, or it was touching the cup. Percent time in contact with either the novel rat or object was calculated by the formula ([total time with target cup (rat or object)/300 s] × 100). Latency (seconds) to approach the novel rat was also recorded (Strzelewicz et al., 2019).

In a separate group of rat dams (*n* = 9), a pup preference test was run using a similar protocol. The purpose of this test was to determine whether dams had a social preference for either SD or EE housed P7 pups, which may have impacted maternal care and later offspring social behavior (Champagne and Curley, 2009; Cromwell, 2011). Dams were assessed on the amount of time they spent exploring alien SD versus alien EE housed pups. One male and one female pup from each housing condition were placed into wire cups situated on opposite sides of an open field arena. The total duration (seconds) that SD and EE dams spent with each housing group across a 10-min period was reported.

### Western blotting

On the morning of P22, a mixture of ketamine/xylazine (150 mg/kg, i.p./50 mg/kg, i.p.) was used to anesthetize dams and their litters. Maternal blood was collected in EDTA coated tubes following a cardiac puncture and spun at 1000 × *g* for 10 min to obtain plasma. Levels of prolactin were determined in undiluted plasma samples using an ELISA (Abcam, catalog #ab272780). Whole hypothalamus was dissected from offspring and dams, frozen on dry ice, and stored at −80°C for future processing. Hypothalamic tissue was later homogenized and the amount of protein in each sample was determined using the BCA assay (Pierce BCA Protein Assay kit; catalog #23227). Protein was mixed with 2× Laemmli sample buffer (Bio Rad Laboratories catalog #1610737) and denatured at 100°C for 5 min. A total of 20 μg of protein was loaded into each well of Mini-Protean gels (Bio-Rad Laboratories, catalog #4568101) and transferred onto nitrocellulose membranes (Bio-Rad Laboratories, catalog #1620147). Membranes were then incubated in a 5% nonfat milk with TBS + 0.05% Tween 20 (TBST) blocking solution for 1 h at room temperature. Following this, they were washed with TBST and incubated in a 1:1000 dilution of CB1 receptor antibody (Abcam catalog #ab259323) in TBS solution overnight at 4°C. The next morning, membranes were washed and incubated in a horseradish peroxidase-conjugated secondary antibody (1:1000, Abcam, catalog #ab131366), made in 1% nonfat milk with TBS, for 1 h at room temperature. Membranes were then washed and immersed in a chemiluminescent substrate (ThermoFisher Scientific, catalog #34580) for 5 min before being scanned with a LI-COR C-DiGit Scanner (model #3600). After imaging, membranes were stripped (ThermoFisher Scientific, catalog #21062) for 15 min at 37°C, followed by blocking in 5% nonfat milk with TBST for 1 h at room temperature. After washing, the membranes were incubated in β-actin (1:1000, ThermoFisher Scientific, catalog #MA515739) for 1 h and imaged again. Densitometry was used to obtain a ratio of CB1/β-actin to quantify protein differences between housing groups.

### Statistical analyses

Statistics were performed using the software package Statistical Software for the Social Sciences (SPSS) version 26.0 (IBM) or GraphPad Prism (version 9.0). The dataset was not powered to evaluate sex-differences so male and female animals were evaluated separately (Ordoñes Sanchez et al., 2021). Two-way repeated measure ANOVAs (housing × time) were used to compare P1 and P10 milk levels of creamatocrit, which is linearly related to the fat concentration and energy content of milk (Lucas et al., 1978; Paul et al., 2015). This statistical analysis was also used to compare the percent of time the dam spent on the nest (P1–P4, P10) across the light and dark phases of the circadian cycle.

A paired samples *t* test was used to evaluate maternal preferences for P7 SD and EE housed pups. One-way ANOVAs were used to evaluate other measures of maternal care and milk composition (e.g., ELISA data) as a function of housing condition. In rare cases of violations to the assumption of normality (Shapiro–Wilk test), Kruskal–Wallis tests were employed (expressed as *Χ^2^*). Offspring behavior was assessed using 2 × 2 (prenatal treatment × postnatal treatment) ANOVAs and LSD *post hoc*s were applied except where there were fewer than three levels, in which case pairwise *t* tests and Levene’s (applied in the occurrence of unequal variances) were used alongside Bonferroni α adjustments. Pearson correlations were analyzed between measures of maternal milk quality and offspring behavior.

Data are graphically expressed as mean ± SEM. The *n_p_*^2^ is also reported as an index of effect size for the ANOVAs (the range of values being 0.02 = small effect, 0.13 = moderate effect, 0.26 = large effect; Miles and Shevlin, 2001).

For the microbiome analyses, composition visualization, α-diversity, and β-diversity analyses were performed with Qiime (v.1.9.1) and statistical comparisons were performed using Kruskal–Wallis tests (Caporaso et al., 2010). To determine taxa that were significantly different between groups, linear discriminant analysis effect size (LEfSe; http://huttenhower.sph.harvard.edu/lefse/) was employed as previously described (Segata et al., 2011; Schellekens et al., 2021). In short, LEfSe creates a model that identifies taxa that are most likely to explain differences between groups through the use of a series of nonparametric tests (Segata et al., 2011).

DESeq2 was used to determine differentially expressed genes based on *p* < 0.05 or FDR adjusted *p* < 0.05, with a FC threshold of 1.3 for RNA-seq.

### Data availability

RNA-seq data have been deposited to GEO (GSE200249). DEGs are included in Extended Data Figures 3-1 and 4-1. All other data are available on request.

### Code availability

There is no code associated with this work.

## Results

### Maternal housing condition affects maternal care

One-way ANOVA showed that EE dams gave birth to larger litters than their SD housed counterparts (SD: 14.0 ± 0.60 vs EE: 16.63 ± 1.05; *F*_(1,14)_ = 4.173, *p* = 0.048, *n_p_*^2^ = 0.252; Fig. 1*D*); all litters were standardized to 10 pups on P1. On P7, a social preference test showed that EE and SD dams investigated all pups to the same extent, regardless of the pups housing origin (*t*_(17)_ = 0.808, *p* = 0.430; Fig. 1*E*).

A repeated measures ANOVA suggested that the percent of time dams spent on the nest did not change as a function of postnatal day across the light and dark periods (*p* > 0.05; Fig. 1*F*,*G*). However, when these two observation periods were collapsed together a significant main effect of postnatal day emerged (*F*_(4,56)_ = 2.74, *p* = 0.037, *n_p_*^2^ = 0.164; Fig. 1*H*) with a greater percentage of time being spent on the nest on P4 (*p* = 0.001), an effect driven by the SD group. Indeed, both the dark and the total light + dark periods combined revealed a significant main effect of housing condition in that SD dams spent a greater percentage of time on the maternal nest than EE dams [dark: *F*_(1,14)_ = 16.987, *p* = 0.001, *n_p_*^2^ = 0.548 (Fig. 1*G*); total light + dark: *F*_(1,14)_ = 9.839, *p* = 0.007, *n_p_*^2^ = 0.830 (Fig. 1*H*)]. This general pattern persisted when the total time (in seconds) dams spent on the nest was summed into a composite score across the postnatal observation days. Again, SD dams were shown to spend more time on the nest than EE mothers [light: *p* > 0.05 (Fig. 1*I*); dark: *Χ^2^*(1) = 7.456, *p* = 0.006 (Fig. 1*J*); total light + dark: *Χ^2^*(1) = 4.864, *p* = 0.027 (Fig. 1*K*)].

The number of maternal nursing postures did not differ as a function of housing in the light phase (*p* > 0.05; Fig. 1*L*). However, the number of total nursing postures observed were significantly lower in EE dams during the dark phase (high arched back: *F*_(1,14)_ = 24.462, *p* = 0.001, *n_p_*^2^ = 0.636; passive/blanket: *X*^2^(1) = 4.498, *p* = 0.034; total nursing: *F*_(1,14)_ = 17.996, *p* = 0.001, *n_p_*^2^ = 0.562; Fig. 1*M*) and in the total combined light + dark phases (high arched back: *F*_(1,14)_ = 10.772, *p* = 0.005, *n_p_*^2^ = 0.435; passive/blanket: *F*_(1,14)_ = 5.296, *p* = 0.037, *n_p_*^2^ = 0.274; total nursing: *X*^2^(1) = 9.289, *p* = 0.002; Fig. 1*N*).

Maternal nest building bouts were significantly increased in SD dams during the light phase (*Χ^2^*(1) = 7.323, *p* = 0.007; Fig. 1*O*). SD dams also licked/groomed their pups more frequently in the dark phase (*F*_(1,14)_ = 22.257, *p* = 0.001, *n_p_*^2^ = 0.614; Fig. 1*P*). However, the total amount of licking and grooming that EE and SD pups received across the total light + dark phases did not differ (*p* > 0.05; Fig. 1*Q*), while increased nest building was sustained in SD dams (*X*^2^(1) = 5.370, *p* = 0.020; Fig. 1*Q*).

During the light phase, maternal self-directed grooming and eating/drinking behaviors were elevated in SD dams (maternal self-grooming: *F*_(1,14)_ = 6.077, *p* = 0.027, *n_p_*^2^ = 0.303; eating/drinking: *F*_(1,14)_ = 4.833, *p* = 0.045, *n_p_*^2^ = 0.257; Fig. 1*R*). SD dams also displayed a higher number of repetitive tail/arm chase behaviors (see Fig. 1*C* for photograph) across the nychthemeron [light: *X*^2^(1) = 6.536, *p* = 0.011; dark: *X*^2^(1) = 6.303, *p* = 0.012 (Fig. 1*S*); total light + dark: *X*^2^(1) = 6.792, *p* = 0.009 (Fig. 1T)] and higher self-grooming levels when the light + dark periods were combined (*F*_(1,14)_ = 9.914, *p* = 0.007, *n_p_*^2^ = 0.415; Fig. 1*T*).

### EE increases lactation quality in terms of the expression of prolactin and triglycerides

**Figure 2.**
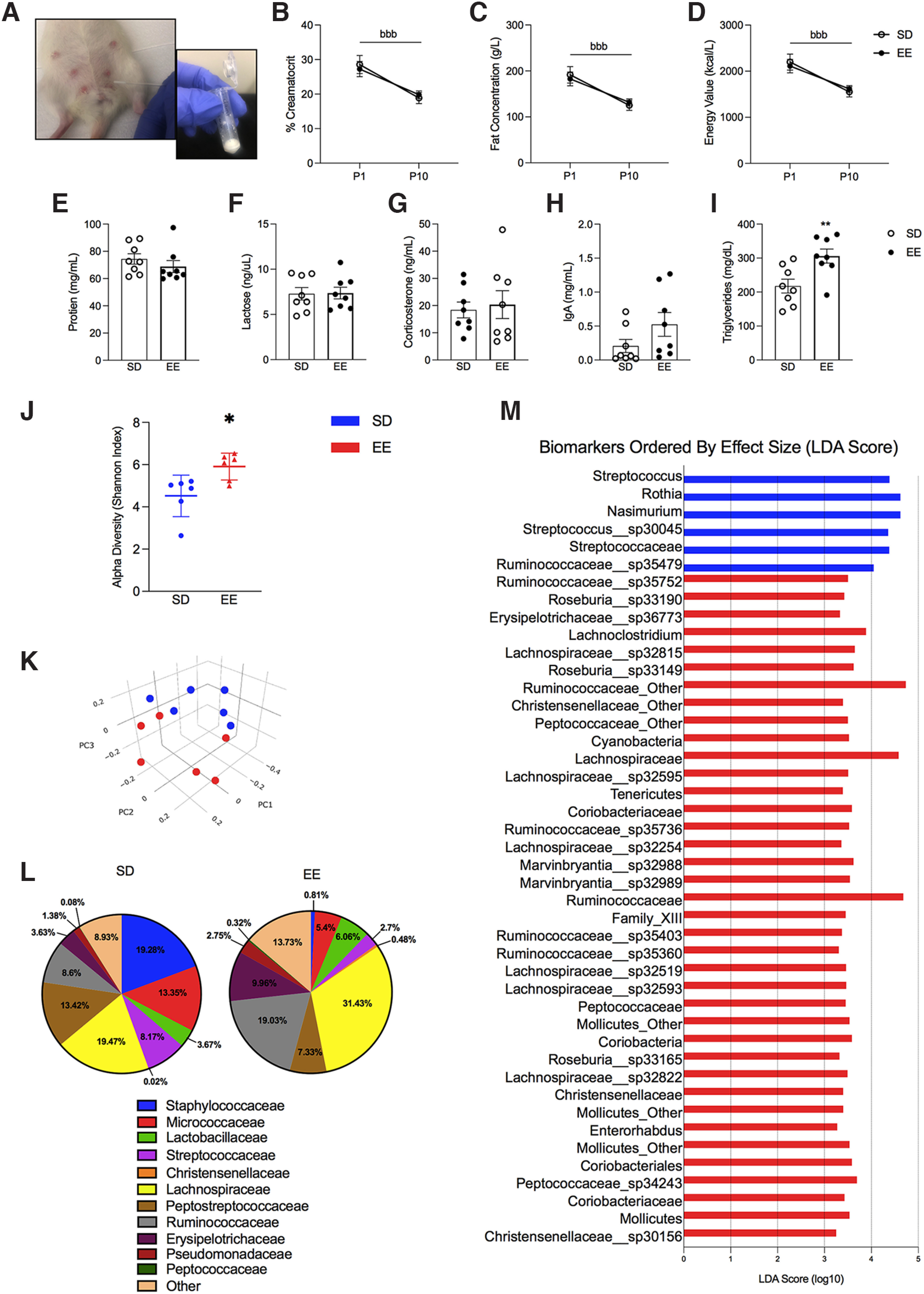
Nutritional profile of milk and microbiome community distribution in EE and SD Sprague Dawley rat dams. ***A***, Photograph depictions of maternal milk collection. Maternal milk concentrations (*n* = 8) of (***B***) % creamatocrit, (***C***) fat (g/l), (***D***) energy value (kcal/l), (***E***) protein (mg/ml), (***F***) lactose (ng/μl), (***G***) corticosterone (ng/ml), (***H***) IgA (mg/ml), and (***I***) triglycerides (mg/dl). Microbiome sequencing (*n* = 6) is demonstrated with (***J***) α diversity along the Shannon index and (***K***) β diversity using principle coordinate analysis (PCoA). This plot was created using the matrix of pair-wise distance between samples determined by Bray–Curtis dissimilarity using unique amplicon sequencing variants. Each dot represents an individual microbial profile. Samples that are closer together are more similar, while samples that are dissimilar are plotted further away from one another. ***L***, Microbial composition of taxonomy in maternal milk at the family level for SD and EE-housed dams. ***M***, Microbiome biomarkers plot. Taxa identified as significantly more abundant in the milk of the housing group where a bar appears; SD mothers (blue bars) and EE mothers (red bars). Significance was determined by LEfSe analysis, which identified taxa with distributions that were statistically significant (*p* < 0.05) and where the effect size (LDA score) was >2. Data are expressed as mean ± SEM; SD: open circles versus EE: closed circles. *p < 0.05; **p < 0.01, SD versus EE; ^bbb^*p* < 0.001, main effect of postnatal day. See Extended Data Figure 2-1.

Given that SD dams spent significantly more time on the nest feeding their litters than EE dams, and that EE dams had higher plasma concentrations of the breastmilk-producing hormone prolactin (*F*_(1,13)_ = 5.69, *p* = 0.034, *n_p_*^2^ = 0.322; Extended Data Fig. 1-1*A*), we evaluated whether there were differences in milk quality between the two housing conditions (see Fig. 2*A* for photograph of milk collection procedure). There was a main effect of postnatal day for % creamatocrit, which is directly proportional to the fat concentration and energy content of milk (Lucas et al., 1978; Paul et al., 2015). These proportionally related measures decreased in both housing groups between P1 and P10 (*F*_(1,14)_ = 23.607, *p* = 0.001, *n_p_*^2^ = 0.001; Fig. 2*B–D*). The ratio of different milk contents changes over the course of lactation in rats, and fat in particular decreases with time (Keen et al., 1981). There were no significant housing effects in the concentration of protein, lactose, corticosterone, or IgA in P10 milk (*p* > 0.05; Fig. 2*E–H*). However, triglyceride levels were significantly higher in the milk of EE compared with SD housed dams (*F*_(1,14)_ = 9.314, *p* = 0.009, *n_p_*^2^ = 0.400; Fig. 2*I*).

10.1523/ENEURO.0148-22.2022.f2-1 Extended Data Figure 2-1 Cladogram of milk biomarkers associated with standard laboratory housing (SD) or EE environments (determined by LEfSe). Diameter of the nodes indicates relative abundance of taxa for SD (green) and EE (red) samples. Placement indicates the classification of taxa, where nodes decrease in rank the closer to the center of the diagram. Download Figure 2-1, TIF file.

### EE increases the microbiome diversity of maternal milk which has a higher abundance of bacterial families related to body weight and energy metabolism

Housing condition also contributed to significant differences in the composition of the milk microbiome (Fig. 2*J–M*). EE milk contained greater species diversity compared with SD milk, as indicated by α diversity along the Shannon index (*X*^2^(1) = 5.77, *p* = 0.016; Fig. 2*J*) and Bray–Curtis dissimilarity measurement of β diversity (*R* = 0.2944, *p* = 0.02; Fig. 2*K*). Please see Extended Data Figures 2-1 and 3-1 for the Cladogram of milk biomarkers and taxonomy heatmap respectively. Overall, LEfSe analysis revealed 44 discriminative taxa between our housing groups, 38 of which were more highly expressed in the milk of EE dams. Specifically, milk from EE dams demonstrated a significantly greater abundance of the phylum *Tenericutes* (LDA effect size = 3.39, *p* = 0.03; Fig. 2*J*). Additionally, *Christensenellaceae* (LDA effect size = 3.39; *p* = 0.007), *Peptococcaceae* (LDA effect size = 3.499; *p* = 0.02), *Coriobacteriaceae* (LDA effect size = 3.59; *p* = 0.006), *Lachnospiraceae* (LDA effect size = 3.32; *p* = 0.007), *Ruminococcaceae* (LDA effect size = 4.73; *p* = 0.02), and *Erysipelotrichaceae* (LDA effect size = 3.33; *p* = 0.02) were higher at the family level (Fig. 2*L*,*M*) in EE mothers. Milk from SD dams had greater levels of *Streptococcaceae* (LDA effect size = 4.38; *p* = 0.02; Fig. 2*L*,*M*).

### RNA-seq identified several maternal milk transcriptomic pathways that were differently affected by housing condition

**Figure 3.**
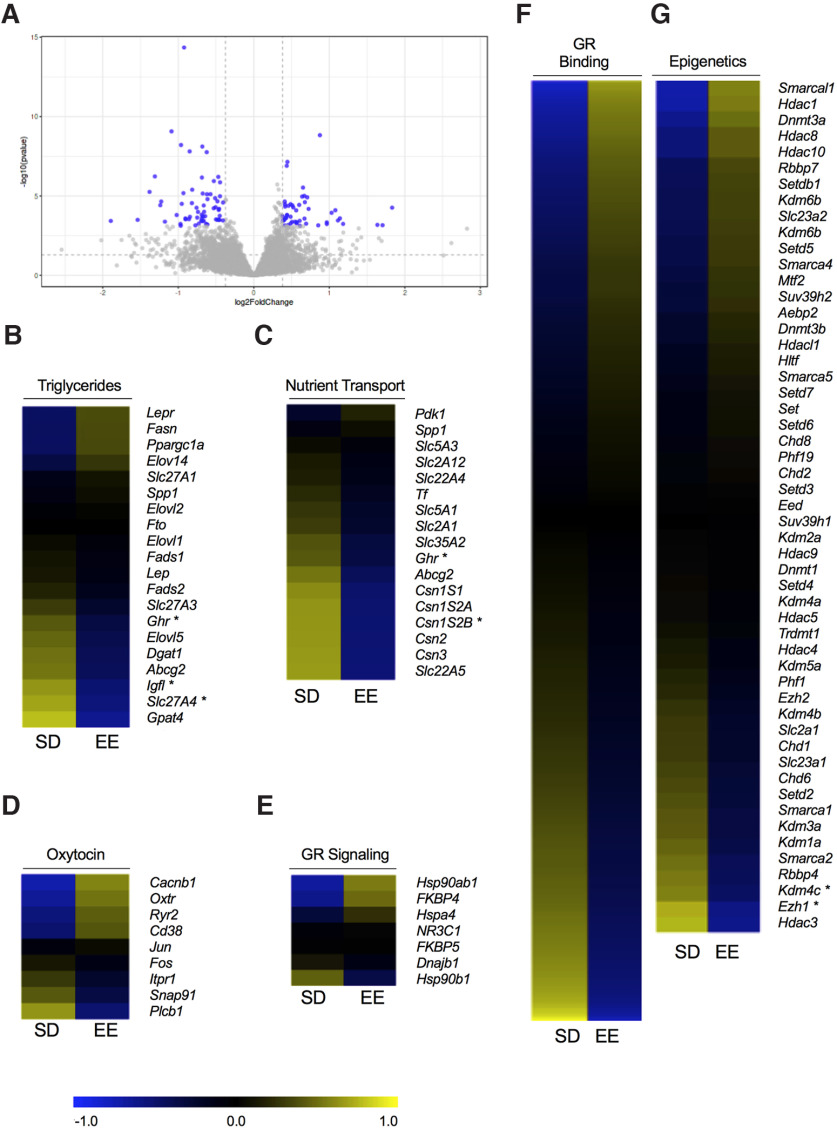
Transcriptomic analyses of P10 milk samples from rat mothers living in EE or standard housing (SD). ***A***, Volcano plot depicting the distribution of 756 genes based on log2 FC and -log10 *p* values. Each gray dot is a gene, and 110 dots highlighted in blue represent genes that displayed the highest magnitude of significance (*p*adj < 0.05, FC > 1.3). Heatmaps of differentially expressed genes related specifically to milk (***B***) triglyceride concentration, (***C***), nutrient transport, (***D***), oxytocin signaling, (***E***) GR signaling, (***F***) GR binding, and (***G***) epigenetics. Gene expression is represented with the log2 transformation of counts recorded with a *z* score based on the average across experimental groups. Data are expressed as *p < 0.05 or **adjusted *p* < 0.05, with FC > 1.3. GR, glucocorticoid receptor. See Extended Data Figures 3-1 and 3-2.

The milk fat layer of breastmilk is a reservoir of epithelial RNA that has only recently begun to be understood using next-generation sequencing (Lemay et al., 2013; Alsaweed et al., 2015). Further, milk fat-derived mRNA contains genes that are involved with neurodevelopment and these genes may be differentially regulated by the maternal environment (Y. Chen et al., 2017). Together, this warrants the broader investigation of maternal milk at the genome-wide level. Based on our identified immunoassay targets and microbiome evaluations, we broadened our analyses to the whole genome context by evaluating transcriptomic profiles of SD and EE milk samples which were characterized using RNA-seq. A total of 756 genes were differently expressed (*p* < 0.05, FC > 1.3) between housing conditions with 110 genes meeting the significant threshold *p*adj < 0.05 (Fig. 3*A*; Extended Data Fig. 3-2). First, we targeted the ontology analysis toward genes that contribute to maternal milk nutrition (Strucken et al., 2015). RNA-seq revealed several differently expressed genes involved with milk triglyceride and nutrient transport downregulated by EE, including *Ghr* (*p* = 0.024, FC = −0.671), *Igf1* (*p* = 0.03, FC = −0.671), *Slc27a4* (*p* = 0.032, FC = −0.447), *Gpat4* (*p* < 0.001, FC = −0.613), and *Cs1s2b* (*p* = 0.009, FC = −0.838; Velmala et al., 1995; Fig. 3*B*,*C*). We broadened the scope of our analyses further to include gene pathways related to oxytocin (Quintana et al., 2019), glucocorticoid receptor (GR) signaling (e.g., events that occur when glucocorticoids bind to the GR receptor; (Oakley and Cidlowski, 2013), GR binding (e.g., genes which bind the GR; Polman, 2012), and epigenetic modifiers (Zhu et al., 2016; Fig. 3*D–G*) given their crucial implication in milk production and offspring development (Zhang et al., 2013; Ozkan et al., 2020). Heatmap clustering showed that EE rats displayed a major downregulation of genes encoding for triglycerides and nutrient transport (Fig. 3*B*,*C*), while genes related to oxytocin and GR (Fig. 3*D*,*E*) signaling were mostly upregulated compared with SD rats. Another cluster of genes related to glutamate/GABA signaling (Gray et al., 2018) were mostly downregulated by EE (Extended Data Fig. 4-1*A*). GR binding genes, epigenetic modifiers, and prolactin-signaling-related genes (Radhakrishnan et al., 2012) were instead equally regulated in both directions (Fig. 3*F*,*G*; Extended Data Fig. 4-1*B*).

10.1523/ENEURO.0148-22.2022.f3-1 Extended Data Figure 3-1 Taxonomy heatmap demonstrating the microbial composition of milk samples from standard laboratory housed (SD) or EE dams at the species level with the top 50 most abundant species identified. The colored bar at the top indicates housing condition (blue = SD, red = EE). Each row represents the abundance for each taxon, with the taxonomy ID shown on the right. Each column represents the abundance for each sample. Download Figure 3-1, TIF file.

10.1523/ENEURO.0148-22.2022.f3-2 Extended Data Figure 3-2 Genes identified with DESeq analysis. List of genes that significantly differed based on a *p*adj < 0.05 and FC > 1.3 as indicated in Figure 3*A*. Download Figure 3-2, DOC file.

### EE offspring had heavier body weights and increased social behavior which was lost by cross-fostering into standard housing in a sex-specific manner

**Figure 4.**
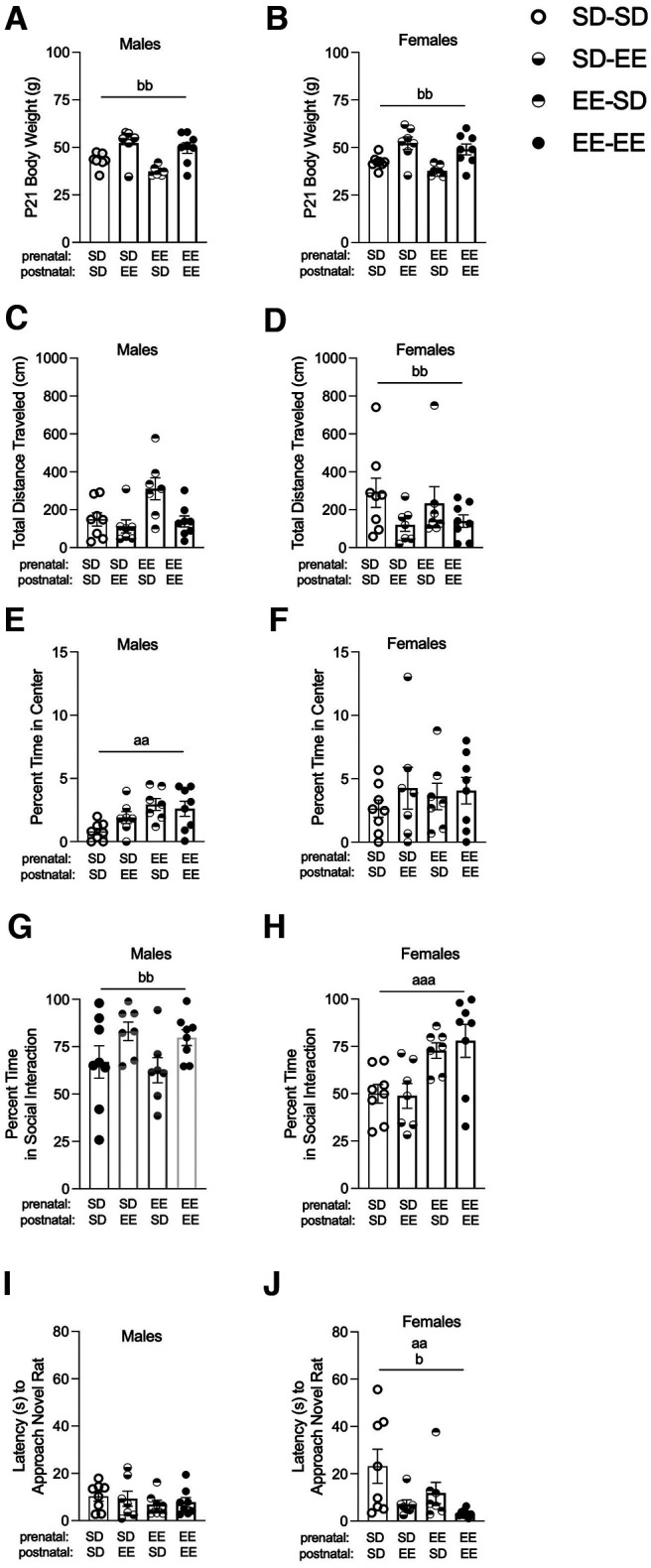
Juvenile offspring physiology and behavior following housing in EE or SD laboratory conditions. Data for (left side) male and (right side) female Sprague Dawley rats for (***A***, ***B***) P21 body weights (grams). ***C***, ***D***, Total distance traveled (centimeters), and (***E***, ***F***) percent of time spent in the center of an open field. ***G***, ***H***, Percent of time spent in social interaction, and (***I***, ***J***) latency (seconds) to approach a novel rat in a social preference test (*n* = 7–8). Data are expressed as mean ± SEM; ^aa^*p* < 0.01, ^aaa^*p* < 0.001, main effect of prenatal experience (SD vs EE); ^b^*p* < 0.05, ^bb^*p* < 0.01, main effect of postnatal experience (SD vs EE). See Extended Data Figures 4-1 and 4-2.

Postnatal housing experience was associated with a significant P21 body weight difference in male and female offspring (males: *X*^2^(1) = 9.562, *p* = 0.002; females: *X*^2^(1) = 11.733, *p* = 0.001; Fig. 4*A*,*B*). Postnatal enrichment housing resulted in significantly higher body weights than SD housing (males SD: 40.43 ± 1.10 g vs EE: 50.86 ± 2.01 g; females SD: 40.191 ± 0.99 g vs EE: 50.50 ± 2.14 g).

10.1523/ENEURO.0148-22.2022.f4-1 Extended Data Figure 4-1 Heatmaps of differentially expressed genes related to glutamate/GABA and prolactin signaling in standard laboratory housed (SD) or EE dams. Gene expression is represented with the log2 transformation of counts recorded with a z-score based on the average across experimental groups. Data are expressed as *p < 0.05 and FC > 1.3, or as ***FC > 1.3 only. Download Figure 4-1, TIF file.

10.1523/ENEURO.0148-22.2022.f4-2 Extended Data Figure 4-2 Pearson correlations between maternal milk quality measures and the behavior of male and female offspring postnatally housed in standard laboratory (SD) or EE environments. Pearson correlations between distance traveled (mm^2^) and % creamatocrit for (***A***) postnatally housed SD males and (***B***) all females regardless of housing. ***C***, Milk energy content (kcal/l) and total distance traveled by all females. Milk fat concentration (g/l; ***D***), % creamatocrit (***E***), and milk energy content (kcal/l; ***F***) correlations for latency to visit the novel rat by postnatally housed SD females. Data are expressed as *p* < 0.05. Download Figure 4-2, TIF file.

There was a main effect of postnatal experience for total distance traveled in that SD female offspring traveled more than EE females (postnatal SD: 262.98 ± 56.69 cm vs postnatal EE: 130.41 ± 22.76 cm; *X*^2^(1) = 3.882 *p* = 0.049; males: *p* > 0.05; Fig. 4*C*,*D*). A main effect of prenatal experience was found for male animals (*X*^2^(1) = 7.618, *p* = 0.006; females: *p* > 0.05; Fig. 4*E*,*F*) on the open field test. Male EE prenatally housed offspring spent (2.76 ± 0.375%) a significantly higher percentage of time in the center of the arena compared with SD prenatally housed males (1.32 ± 0.285%), although the overall times were quite low.

For the percent of time spent in social interaction, there was a main effect of postnatal experience for male offspring in that postnatal enrichment housing increased social interest (postnatal SD: 64.88 ± 5.36 vs postnatal EE: 81.34 ± 3.09; *F*_(1,26)_ = 6.840, *p* = 0.015, *n_p_*^2^ = 0.208; Fig. 4*G*); the enrichment effect was blocked for animals cross-fostered into SD housing (Fig. 4*G*). For females, the EE prenatal experience increased social engagement level (prenatal SD: 49.44 ± 3.88 vs prenatal EE: 75.54 ± 4.91; *F*_(1,26)_ = 16.140, *p* = 0.001, *n_p_*^2^ = 0.383; Fig. 4*H*). Both prenatal (SD = 15.66 ± 4.37 and EE = 7.30 ± 2.34; *X*^2^(1) = 8.073, *p* = 0.004) and postnatal (SD = 17.90 ± 4.48 and EE = 5.06 ± 1.02; *X*^2^(1) = 4.047, *p* = 0.044;) enrichment experience decreased the latency for female offspring to approach the novel rat (Fig. 4*I*,*J*).

The relationship between measures of maternal milk quality and offspring behavior were assessed using Pearson correlations. In postnatally housed SD males, % creamatocrit was negatively associated with total distance traveled (*r* = −0.597, *p* = 0.019; Extended Data Fig. 4-2*A*). Percent creamatocrit was positively correlated with total distance traveled (*r* = 0.369, *p* = 0.049; Extended Data Fig. 4-2*B*), while milk energy content (*r* = −0.369, *p* = 0.049; Extended Data Fig. 4-2*C*) was negatively associated with this measure in female offspring regardless of housing condition. In postnatally housed SD female offspring, fat (*r* = −0.577, *p* = 0.024) and % creamatocrit (*r* = −0.591, *p* = 0.020) were negatively correlated (Extended Data Fig. 4-2*D*,*E*) and milk energy content (*r* = 0.591, *p* = 0.020) was positively correlated with the latency to approach the novel rat (Extended Data Fig. 4-2*F*).

We assessed the expression of the endocannabinoid receptor CB1 in the hypothalamus of mothers and their offspring given its role in maternal behavior (Schechter et al., 2012) and in the development of social behavior (Argue et al., 2017). While there were no housing-associated differences in CB1 in the offspring (*p* > 0.05; Extended Data Fig. 5-1*A–C*), EE dams exhibited significantly greater expression of CB1 compared with SD dams (*X*^2^(1) = 4.45, *p* = 0.035; Extended Data Fig. 5-1*D*).

**Figure 5.**
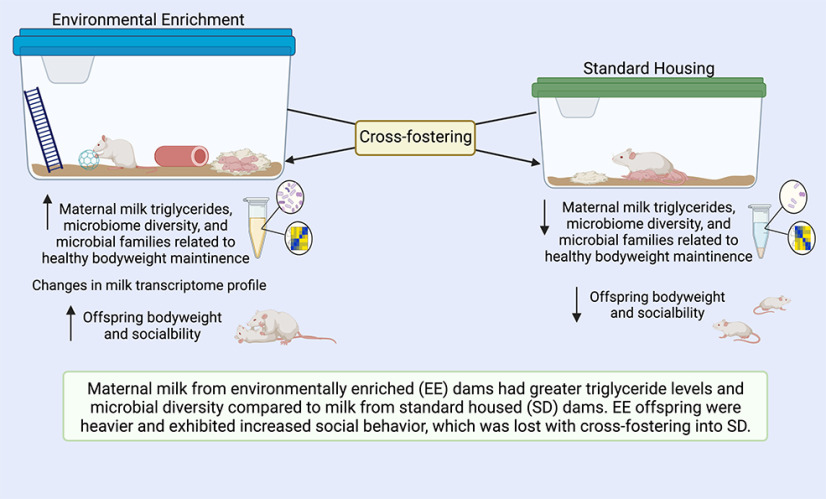
Summary of findings and proposed mechanisms.

10.1523/ENEURO.0148-22.2022.f5-1 Extended Data Figure 5-1 Hypothalamic CB1 and densitometric ratios for offspring and their dams housed in standard laboratory (SD) or EE environments. Males (***A***), females (***B***), and mothers (***C***) housed in SD (open circles) or EE (closed circles) housing conditions. Data are expressed as mean ± SEM, *n* = 7–8; *p < 0.05, main effect of housing (SD vs EE). Download Figure 5-1, TIF file.

## Discussion

In the present study we demonstrate that EE can change the nutritional and microbial profiles of maternal milk in addition to affecting maternal behavior and offspring development. Our results shed light on the multidimensional impact that EE confers on the prenatal and neonatal environment and calls attention to the implications of laboratory housing in developmental animal research. Overall, we observe that cross-fostering blocks the beneficial effects of EE housing on (male) offspring social behavior and (male and female) body weights, implicating maternal milk quality as a potential mechanism (Fig. 5). While EE was associated with greater bodyweights and sociability in both sexes, the finding that EE dams spent less time on the nest may seem contradictory. However, laboratory rats will spend time away from the nest when given the opportunity, which facilitates more efficient nursing (Ratuski and Weary, 2021).

A reduced number of nursing posture displays, coupled with a general increase in time away from offspring, may suggest that EE dams are more efficient at nursing while on the nest. This is further supported by EE-associated increases in circulating prolactin, which promotes milk biosynthesis (Cregan et al., 2002). SD dams may instead need to shift between nursing postures more frequently to maintain an active arched back posture, given the extended periods they spend nursing their young. This may artificially increase the amount of nursing postures they display, alongside the timing of maternal behavior observations (e.g., scoring more frequently in the light or dark phases) which can modulate nursing activity level (Peña and Champagne, 2013). During our maternal care observations, we did not plan to quantify “press posture” (Cramer et al., 1990; Gaskill and Pritchett-Corning, 2015; Ratuski and Weary, 2021) but we subjectively noted its presence in SD dams when it was noticed during our study. A representative photograph of this posture can be found in Figure 1*C*. We did not observe this behavior in any EE dams. Notably, EE and SD dams demonstrated similar amounts of pup licking and grooming despite the differences in time spent on the nest, further supporting the idea that EE dams are more efficient with their care (Sale et al., 2004; Welberg et al., 2006; Connors et al., 2015; Strzelewicz et al., 2019). Although not assessed here, enhanced laboratory caging complexity has previously been shown to reduce maternal care unpredictability (Knop et al., 2020), which could help explain differences in maternal care efficiency. In addition, higher triglyceride levels in EE milk may compensate for the reduced time on the nest, while SD mothers spend more time nursing to make up for lower milk fat content. Alternatively, SD dam milk quality is reduced because they cannot meet the physiological demands of constant nursing. This is supported by the greater offspring bodyweights of pups that received postnatal EE compared to SD care.

Greater bodyweights are generally associated with better health in wild animals (Barnett, 1958a,b; Barnett and Dickson, 1984), especially at the age of weaning (Storey and Snow, 1987; Huber et al., 2002). Moreover, heavier adult male wild rats were better integrated socially within their colony (Barnett and Dickson, 1984). These results underscore the translational relevance of EE used in the present study. The increased bodyweights of EE pups may in part be explained by differences in maternal milk quality. Although we did not find differences in the amount of lactose, protein, or IgA in the milk of EE and SD dams, we did find greater triglyceride levels in EE milk at P10. In support of this finding, Y. Chen et al. (2017) observed that mouse pups fostered to dams with greater levels of milk triglycerides weighed significantly more at P12 than pups that were fostered to control mice. Interestingly, other studies have also demonstrated the role of increased fat consumption on milk triglyceride levels (Mohammad et al., 2014; Ward et al., 2021). Notably, our EE and SD dams did not differ in their total eating bouts or bodyweights (Extended Data Fig. 1-1*B*,*C*), suggesting that EE positively contributes to milk triglycerides through a different mechanism.

We further supported our finding regarding increased triglycerides in EE milk with microbiome sequencing. Bacteria in maternal milk prime the infant gastrointestinal tract, which can affect its maturation and the future metabolism of nutrients and bacteria (reviewed in depth in Macpherson et al., 2017). Results of microbiome sequencing demonstrated a robust effect of EE on bacterial diversity in milk samples. For example, milk from EE dams had a significantly higher abundance of *Christensenellaceae*, *Peptococcaceae*, *Lachnospiraceae*, *Ruminococcaceae*, and *Erysipelotrichaceae*, all of which can directly influence lipid metabolism and body mass index through their manipulation of short-chain fatty acids (Greiner and Bäckhed, 2011; Bridgewater et al., 2017; Waters and Ley, 2019; Vacca et al., 2020). Furthermore, milk from EE dams had greater levels of *Coriobacteriaceae,* which contributes to lipid metabolism (Liu et al., 2018), as well as glucose and steroid metabolism in the gut (Clavel et al., 2014). Although increased *Coriobacteriaceae* have been found in the ceca of mice exposed to stress (Bangsgaard Bendtsen et al., 2012), it is unlikely our EE dams were significantly more stressed than our SD dams given the lack of significant differences in milk corticosterone and dam body weights. In addition to finding enhanced microbiome diversity in milk from EE dams, microbiome sequencing revealed significantly greater levels of taxa from the *Streptococcaceae* family in SD dams. Excess expression of this bacterial family in the infant gut has been tied to GI-related issues like dyspepsia and rotavirus infections (S. Y. Chen et al., 2017; Sohail et al., 2021) which can negatively impact infant growth. Thus, EE may enhance offspring development by altering the microbiome profile of milk that not only promotes the colonization of healthy bacteria, but also discourages exposure to potentially harmful bacteria that can hinder offspring development. While there is a large literature dedicated toward uncovering the role of the built environment (e.g., manufactured materials) in contributing to host microbiology (Gilbert and Stephens, 2018), more work is needed to understand how these environmental factors contribute more specifically to the microbiology of maternal milk and the subsequent impacts on offspring.

In addition to microbial differences, milk from SD and EE dams demonstrated significant differences in their transcriptomic profiles. Although several of the reported genes related to milk triglyceride and nutrient transport were significantly downregulated in EE dams, despite being lipolytic, continuous release of hormones such as Igf1 has been shown to reduce lactation and offspring body weight over time in rats (Lékó et al., 2017). Therefore, the reduced expression of these genes in the milk from EE dams may be reflective of more effective endocrinological signaling that allows the mother to return to a basal state through negative feedback mechanisms. In SD dams however, the constant activation of genes such as *Ghr* may be from spending more time on the nest, which compromises milk quality over the course of lactation. Other genes that were differentially regulated between SD and EE milk samples were those related to GR binding, epigenetic modifications, and glutamate/GABA signaling, all of which have considerable implications in offspring brain development and behavior. Together, the results from RNA-seq offer new insight into how housing condition uniquely affects the milk transcriptomics. These data open a door to potential genomic targets and broader networks that are implicated in EE models. The future validation of individual genes within these identified pathways through the use of additional techniques like qPCR, will be advantageous in delineating the directionality (i.e., upstream vs downstream) of these networks, and may help reconcile some of the differences observed in offspring physiological and behavioral outcomes.

Our observation of increased sociability in EE offspring is in line with several previous studies (Morley-Fletcher et al., 2003; Peña et al., 2006; Schneider et al., 2006; Connors et al., 2014), although these studies used EE to rescue social impairments following early life insults. In saline treated control rats, EE was associated with greater time spent in social interaction (Connors et al., 2014), suggesting that EE is not just protective but can promote sociability. We expand on these findings by revealing sex-specific and time-specific effects of EE on social preference behavior in healthy offspring. Research assessing the effects of EE on healthy populations is warranted and positively contributes to the translatability of this housing model (Kentner et al., 2019b, 2021). Cross-fostering pups between housing conditions after parturition revealed that prenatal enrichment increased sociability in females and postnatal enrichment increased sociability in males. While studies of nonhuman primates have been more consistent in identifying lactation-associated sex differences in offspring behavior (Hinde, 2009; Hinde et al., 2015; Dettmer et al., 2018), these studies also benefit from larger sample sizes. Although our group sizes were modest, the use of larger samples in the rodent literature will be useful in establishing the magnitude of our observed sex differences (Silk et al., 2005).

In addition to differences in maternal care, it is plausible that consumption of different milk microbiome profiles may have directly influenced offspring behavior. Although the relationship between the gut microbiome and social behavior is well established (Archie and Tung, 2015), less is understood about how these bacterial taxa exert their effects on brain development and function once colonized in gut. While interactions between the gut microbiome and brain activity are one driving mechanism in rodent social behavior (Wu et al., 2021), without an assessment of offspring duodenum taxa, we cannot conclude that differences in the gut microbiome profile of our SD and EE offspring were involved in our housing-associated alterations in social behavior.

We examined the expression of hypothalamic CB1 in our offspring since this receptor mediates social development in both males and females (Schechter et al., 2012; Argue et al., 2017). Moreover, CB1 in the neonatal brain is associated with the initiation of suckling (Fride et al., 2001). Blocking CB1 function in lactating dams can impair maternal care, prevent pup weight gain, and modulate the social development of offspring (Schechter et al., 2012). Together, these studies suggest that both maternal and offspring CB1 signaling are crucial elements to the lactational period and postnatal offspring development. Although offspring hypothalamic CB1 expression did not differ between our housing groups, EE dams had a significantly greater expression of hypothalamic CB1 compared with SD dams. This supports the involvement of CB1 in the EE-associated changes in maternal nurturance and milk quality, although it is unknown whether hypothalamic CB1 activity modulates lactational quality directly. Nonetheless, these findings demonstrate the importance of considering maternal brain physiology in the mediation of offspring developmental outcomes.

While the physiological mechanisms that contribute to time-specific and sex-specific differences in behavior among EE offspring need further elucidation, the present results shed light on maternal care and maternal milk quality as pathways of interest for future studies to explore. Recent work has shown running wheel activity to alter milk quality in terms of specific inflammatory molecules such as leukocyte inhibitory factor, CXCL1, and CXCL2; however maternal care was not affected (Taki et al., 2020). This suggests there may be something special in the qualia of the EE condition that extends beyond increased physical activity in terms of its contribution to maternal-neonatal interactions. In addition, prenatal measures, such as the hormonal milieu during pregnancy, may also be of interest. EE dams gave birth to larger litters, and this may indicate that the benefits of EE can manifest far before parturition. Presumably, the compounding effects of maternal behavior and milk quality contribute to expedited plasticity in the developing brain (Cancedda et al., 2004; Baroncelli et al., 2010).

In conclusion, the efficaciousness of the “gold standard” housing cages in animal research has recently been called into question (Olsson and Dahlborn, 2002; Prendergast et al., 2014; Kentner et al., 2019b, 2021). Results of the present study bolster the argument that EE housing conditions encapsulate a more naturalistic environment than SD, especially with regard to maternal behavior and development (Connors et al., 2015; Ratuski and Weary, 2021; Zhao et al., 2021). Given that EE animals do not display more phenotypic variability compared with those in standard housing (Wolfer et al., 2004; Würbel, 2007; André et al., 2018; Kentner et al., 2021), this work suggests that increasing environmental heterogeneity in laboratory settings may help increase translational relevance. Overall, we expand on previous studies that have highlighted the beneficial effects of EE on laboratory rodents by demonstrating the multifaceted impact of EE on maternal care, physiology, and offspring social behavior. These results suggest that an enriched maternal environment contributes to notable, long-term changes in offspring development by promoting more efficient maternal behavior and improved milk quality. Rodent models of breastfeeding are advantageous in teasing apart the mechanisms by which this fascinating substance exerts its influence on brain development and behavior.

## Synthesis

Reviewing Editor: Aniko Korosi, Universiteit van Amsterdam Decisions are customarily a result of the Reviewing Editor and the peer reviewers coming together and discussing their recommendations until a consensus is reached. When revisions are invited, a fact-based synthesis statement explaining their decision and outlining what is needed to prepare a revision will be listed below. The following reviewer(s) agreed to reveal their identity: Silvie Ruigrok.

Reviewer 1

This is a really exciting research program that moves forward the field. There has been decades of long-term work on maternal environment and behavioral care of pups and separately work on milk composition and infant development, but to date little research in rodents has integrated these paradigms. The present study does so elegantly and provides an initial foundation for further explorations at this interface. AND will have important implications for laboratory animal research and intergenerational effects. The authors may also want to look at Stone, Anita Iyengar, and Karen Lisa Bales. “Intergenerational transmission of the behavioral consequences of early experience in prairie voles.” Behavioural processes 84, no. 3 (2010): 732-738. that shows what seems to be the MINOR-EST of differences in early life experience having large long term effects in lab rodents.

I have a few comments that I think should be addressed in the MS:

Statistics:

There is A LOT going on in this paper, especially with relatively small sample sizes- many many many outcomes tested. I think given the careful design and early exploratory nature of this work, that this is not catastrophic, but I think a bit more caution in recognizing the small sample sizes have the potential to overestimate effect sizes of sex differences (see Figure 1 Silk, Joan B., Elisabeth Willoughby, and Gillian R. Brown. “Maternal rank and local resource competition do not predict birth sex ratios in wild baboons.” Proc. R. Soc. B 272 (2005): 859-864.) There is an fascinating, but small literature, on sex-differentiated milk composition and effects of milk in macaques, humans, & marsupials that typically have larger sample sizes that could be cited/leveraged in interpretations in the present manuscript.

Litter sex ratios- int he methods I couldn’t find info on the litter sex ratio gestated, the ratio when limited to 10 pups, and the ratio in cross-fostering. Koskela and colleagues found that sex-ratio influenced milk volume in bank voles so it would be reassuring to know that litter manipulations were not ALSO a radical alteration of the litter sex ratio from pre-natal to post-natal conditions (see Koskela, Esa, Tapio Mappes, Tuuli Niskanen, and Joanna Rutkowska. “Maternal investment in relation to sex ratio and offspring number in a small mammal-a case for Trivers and Willard theory?.” Journal of Animal Ecology 78, no. 5 (2009): 1007-1014.)

SD vs EE microbial profiles- the MS is written as though psychological and behavioral profile differences between the SD & EE conditions are necessarily driving differences in microbial communities- could it also be that the physical spaces/compositions of the two conditions also contribute- there is a rich literature of the microbiome of the built environment that can’t necessarily be ruled out (although the animals’ experiences in the environments are without doubt the key driver).

The paper is clear, well-written and has clear empirical results. It is my own bias that wanted more discussion of life-history and early life organization- I don’t think such things are essential in the present manuscript, but I would encourage the authors to look into those areas of literature as related to the behavioral biology of milk.

Reviewer 2

In the current study, the authors show that environmental enrichment (EE) during pregnancy and lactation increases nest size, and alters maternal care and the maternal milk composition. More specifically, milk of EE dams had higher triglyceride levels and greater microbiome diversity. Offspring of EE dams had higher body weights and EE affected offspring social behavior in a sex- and time-specific manner. Little is known about environmental effects on milk quality. This work is therefore novel and of interest to the field. However, I have several concerns that need to be addressed before publication is possible.

In the current study, the authors show that environmental enrichment (EE) during pregnancy and lactation increases nest size, and alters maternal care and the maternal milk composition. More specifically, milk of EE dams had higher triglyceride levels and greater microbiome diversity. Offspring of EE dams had higher bodyweights and EE affected offspring social behavior in a sex- and time-specific manner. Little is known about environmental effects on milk quality. This work is therefore novel and of interest to the field. However, I have several concerns that need to be addressed before publication is possible.

Significance statement

- Sentence 52-54: not directly tested in the current study (neither the overfeeding nor if the milk quality directly affects neurodevelopmental outcomes). I suggest the authors stay closer to their own study/results for the significance statement.

Introduction

- Sentence 65-68: since the evidence for this statement comes from the intestine, please remove the word ’directly’ in sentence 66.

- Please provide more introduction into the milk microbiome and why it is interesting to look at this with regard to EE and/or offspring behavior.

Methods

- Sentence 128-131: How many enrichment toys each time?

- How much time before breeding were dams of the EE group housed with enrichment?

- Maybe move the part on the maternal care observations more forward, since it’s the first data you’re discussing in the result section. Please also provide more information on the scoring and analysis of maternal behavior (also see below).

- To better understand the maternal care observation periods, what were the times light on and off in the animal facility?

- Was behavior (OF and social behavior) done in light or dark phase?

- From how many nests are the offspring coming (data figure 4)? Is there tested for a contributing effect of nest (e.g. with mixed model analysis)?

Results/discussion

- Figure 1F-H: I think it would make more sense to show the time on the nest as a percentage of the observation time.

- Sentence 346-350, regarding the press posture. If this is something you scored, please quantify, if not I suggest to remove this.

- Figure 1L-T: how did you exactly quantify this, is this an accumulation of all the observation periods over all the days? How did the scoring exactly happen?

- Did the authors look at maternal care predictability? As done in https://onlinelibrary.wiley.com/doi/full/10.1111/gbb.12655. This could be interesting to add.

- Sentence 351: please change to ’The number of maternal nursing postures’.

- Sentence 373-374: the title suggests there were effects of EE on creamatocrit, but the authors do not actually seem to find that (fig 2).

- Figure 4: in my opinion this experiment is too unspecific. Where is the RNA in milk expected to come from? What cell types are present in milk? The authors should provide more clarification on this or otherwise I suggest to remove the experiment from the study.

- Sentence 430 and 432: ’extended data figure 4’.

- Sentence 434: EE offspring instead of enriched offspring.

- Figure 4: Since there are several sex differences in the offspring, I do not think it adds much to report the males and females together as well (Fig 4C, F, I, L, O).

- Please carefully check if F/ X2 values (e.g. sentence 463: if an ANOVA is used, why is there a X2 instead of F value?)

- Sentence 461 and 579: saline dams?

- To get a better understanding of how milk quality could impact the offspring, are there any significant correlations between milk values, e.g. triglyceride levels, and offspring behavior?

- Sentence 469-471: this is somewhat overstated in my opinion. The data presented in the current study suggests a role for maternal milk, but does not implicate a driving mechanism.

- Sentence 489-492 is unclear.

- Sentence 505-507: where is this data?

- Sentence 537: is there any evidence that time on nest may affect milk Ghr levels?

- Sentence 567-568 is unclear.

- In the conclusion paragraph the running comes a bit sudden, the comparison running/EE has not been discussed before in this study. Maybe the conclusion paragraph is not the place to mention this for the first time.

- Extended figure 2: please increase the font of the legend.
