# Supplementary figures and images for: Milking It for All It’s Worth: The Effects of Environmental Enrichment on Maternal Nurturance, Lactation Quality, and Offspring Social Behavior
Source: eNeuro. 2022 Aug 25;9(4):ENEURO.0148-22.2022. doi: 10.1523/ENEURO.0148-22.2022 (PMC9417599; doi:10.1523/ENEURO.0148-22.2022)

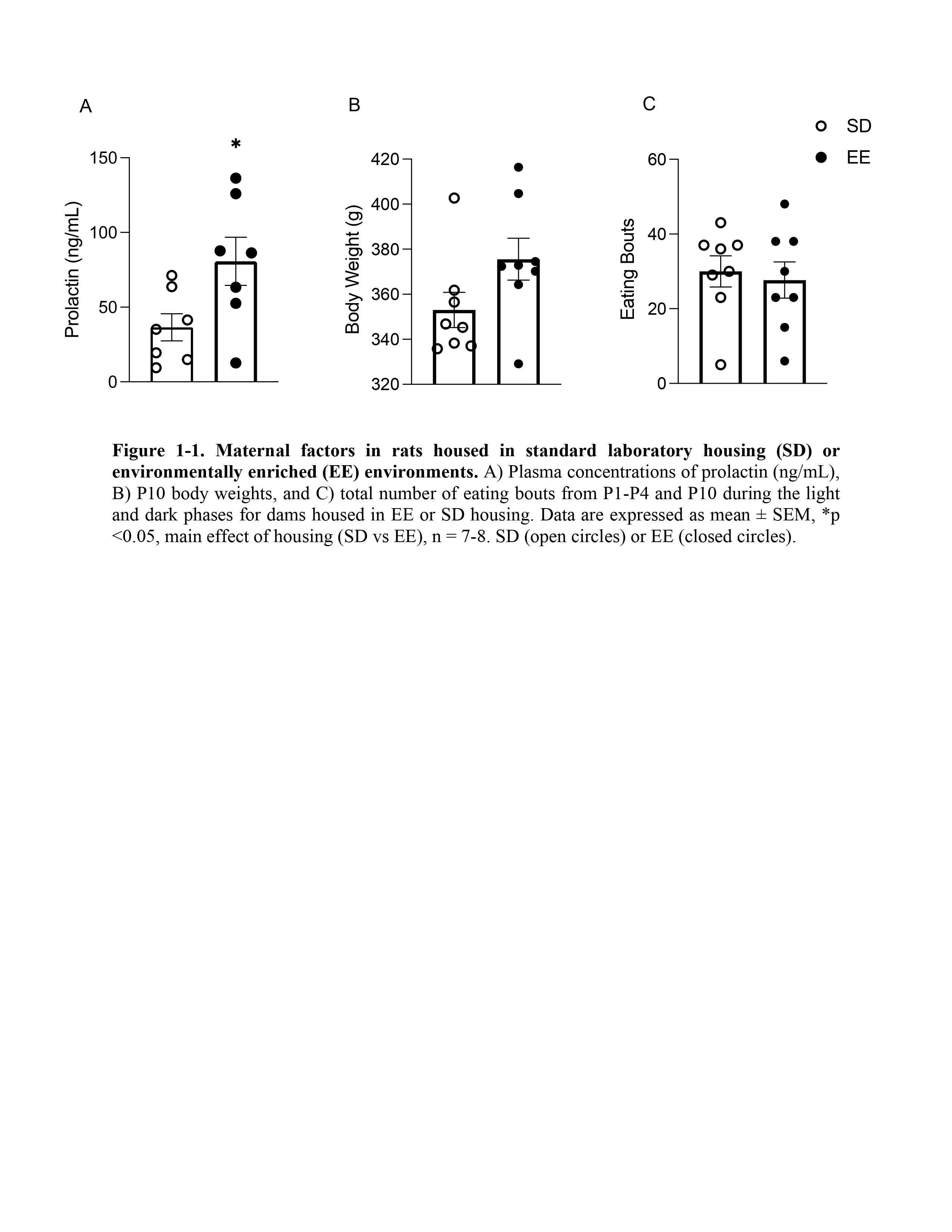

Supplement: Extended Data Figure 1-1 — Maternal factors in rats housed in standard laboratory housing (SD) or EE environments. Plasma concentrations of prolactin (ng/ml; A), P10 body weights (B), and total number of eating bouts (C) from P1 to P4 and P10 during the light and dark phases for dams housed in EE or SD housing. Data are expressed as mean ± SEM; *p < 0.05, main effect of housing (SD vs EE), n = 7–8. SD (open circles) or EE (closed circles). Download Figure 1-1, TIF file. [file enu-eN-NWR-0148-22-s03.tif]

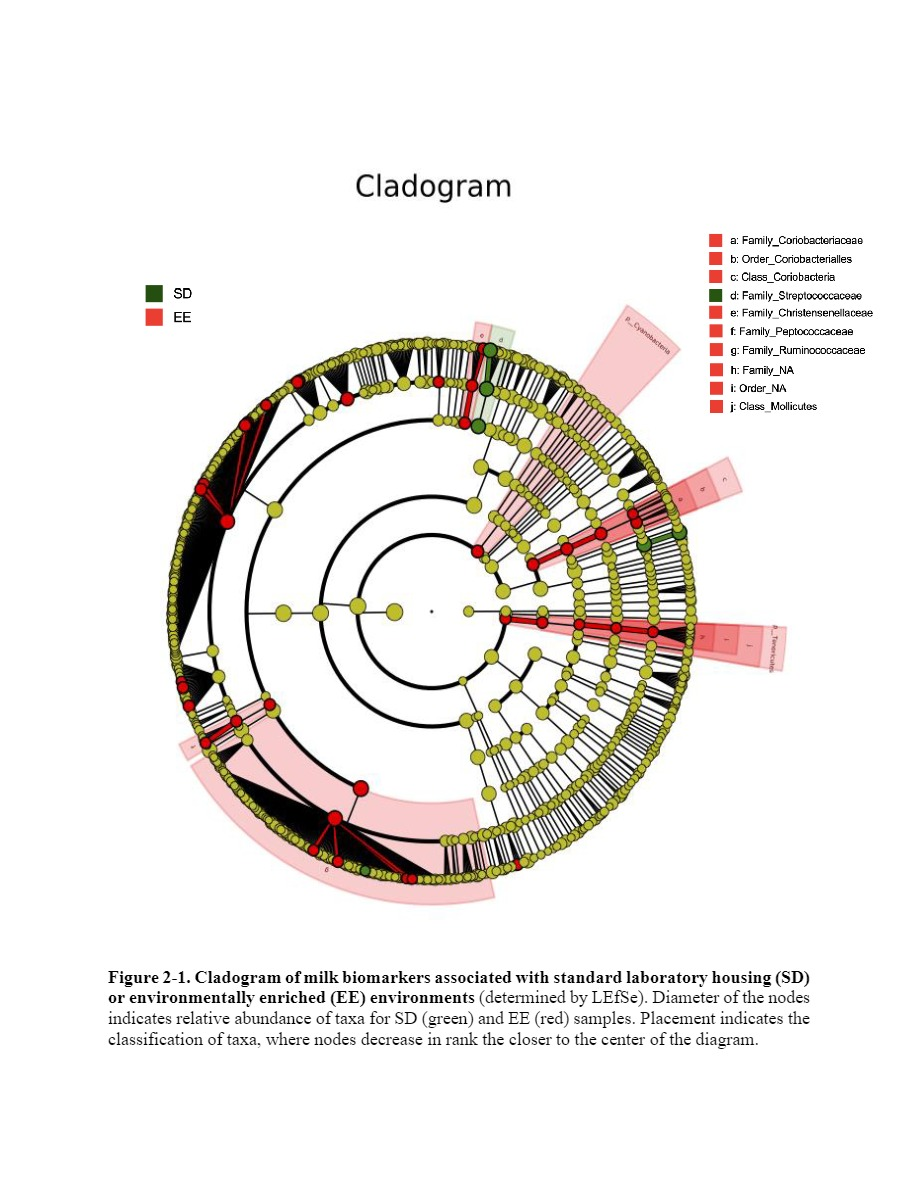

Supplement: Extended Data Figure 2-1 — Cladogram of milk biomarkers associated with standard laboratory housing (SD) or EE environments (determined by LEfSe). Diameter of the nodes indicates relative abundance of taxa for SD (green) and EE (red) samples. Placement indicates the classification of taxa, where nodes decrease in rank the closer to the center of the diagram. Download Figure 2-1, TIF file. [file enu-eN-NWR-0148-22-s04.tif]

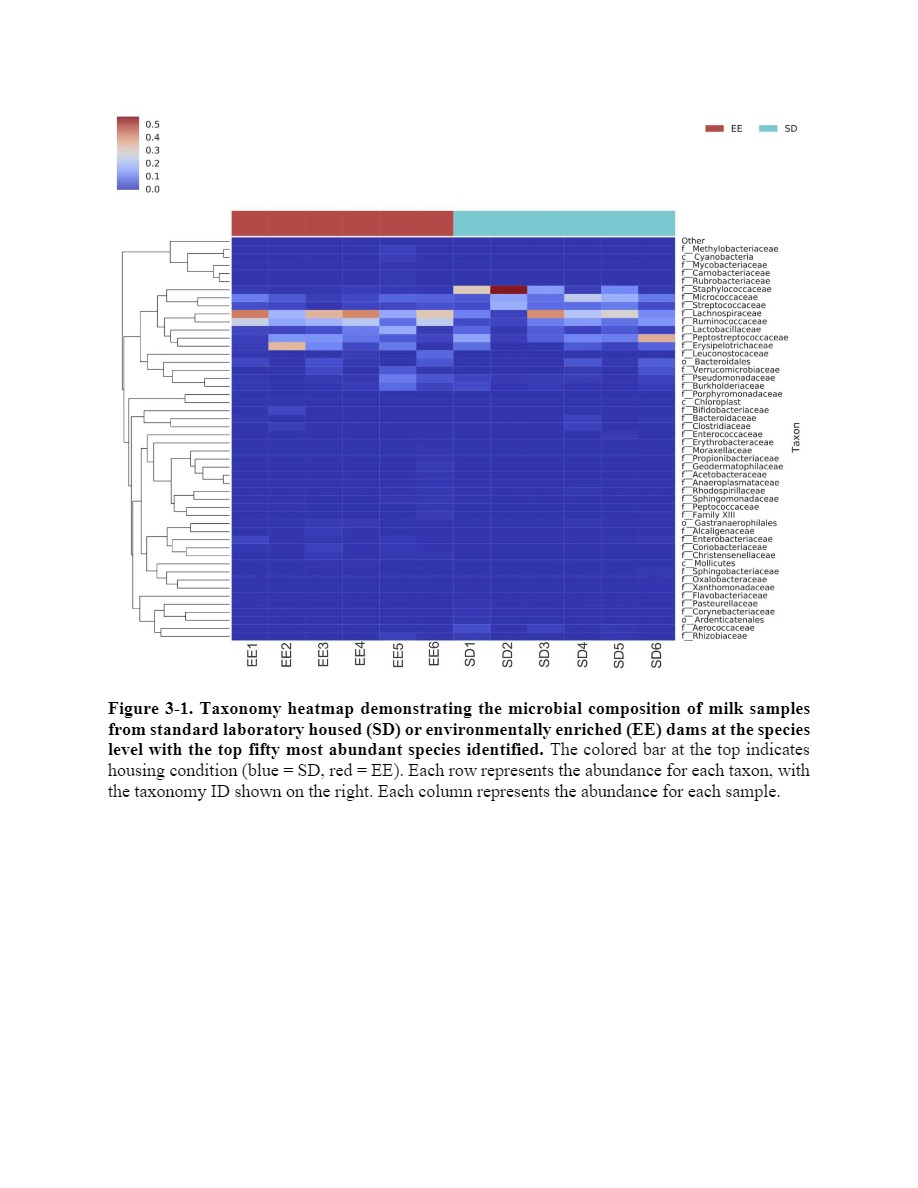

Supplement: Extended Data Figure 3-1 — Taxonomy heatmap demonstrating the microbial composition of milk samples from standard laboratory housed (SD) or EE dams at the species level with the top 50 most abundant species identified. The colored bar at the top indicates housing condition (blue = SD, red = EE). Each row represents the abundance for each taxon, with the taxonomy ID shown on the right. Each column represents the abundance for each sample. Download Figure 3-1, TIF file. [file enu-eN-NWR-0148-22-s05.tif]

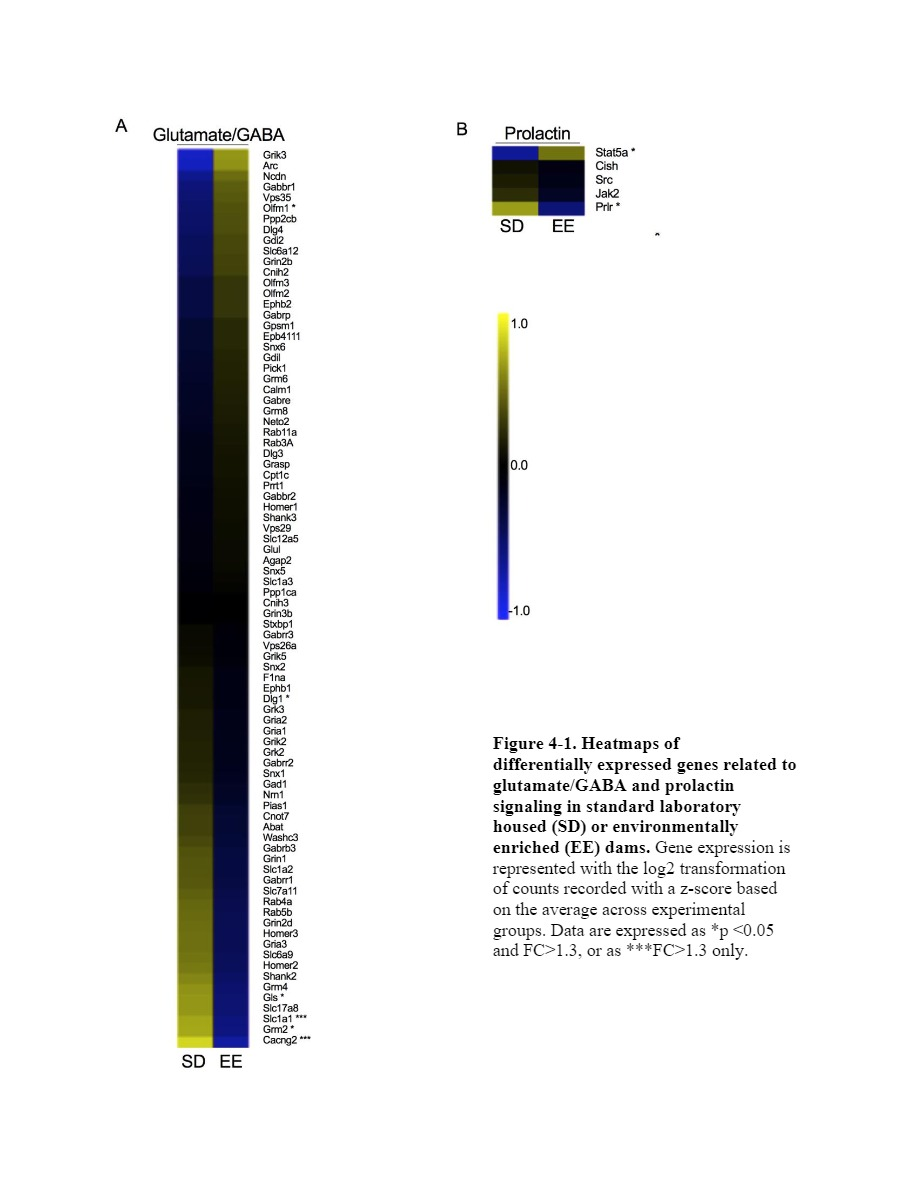

Supplement: Extended Data Figure 4-1 — Heatmaps of differentially expressed genes related to glutamate/GABA and prolactin signaling in standard laboratory housed (SD) or EE dams. Gene expression is represented with the log2 transformation of counts recorded with a z-score based on the average across experimental groups. Data are expressed as *p < 0.05 and FC > 1.3, or as ***FC > 1.3 only. Download Figure 4-1, TIF file. [file enu-eN-NWR-0148-22-s06.tif]

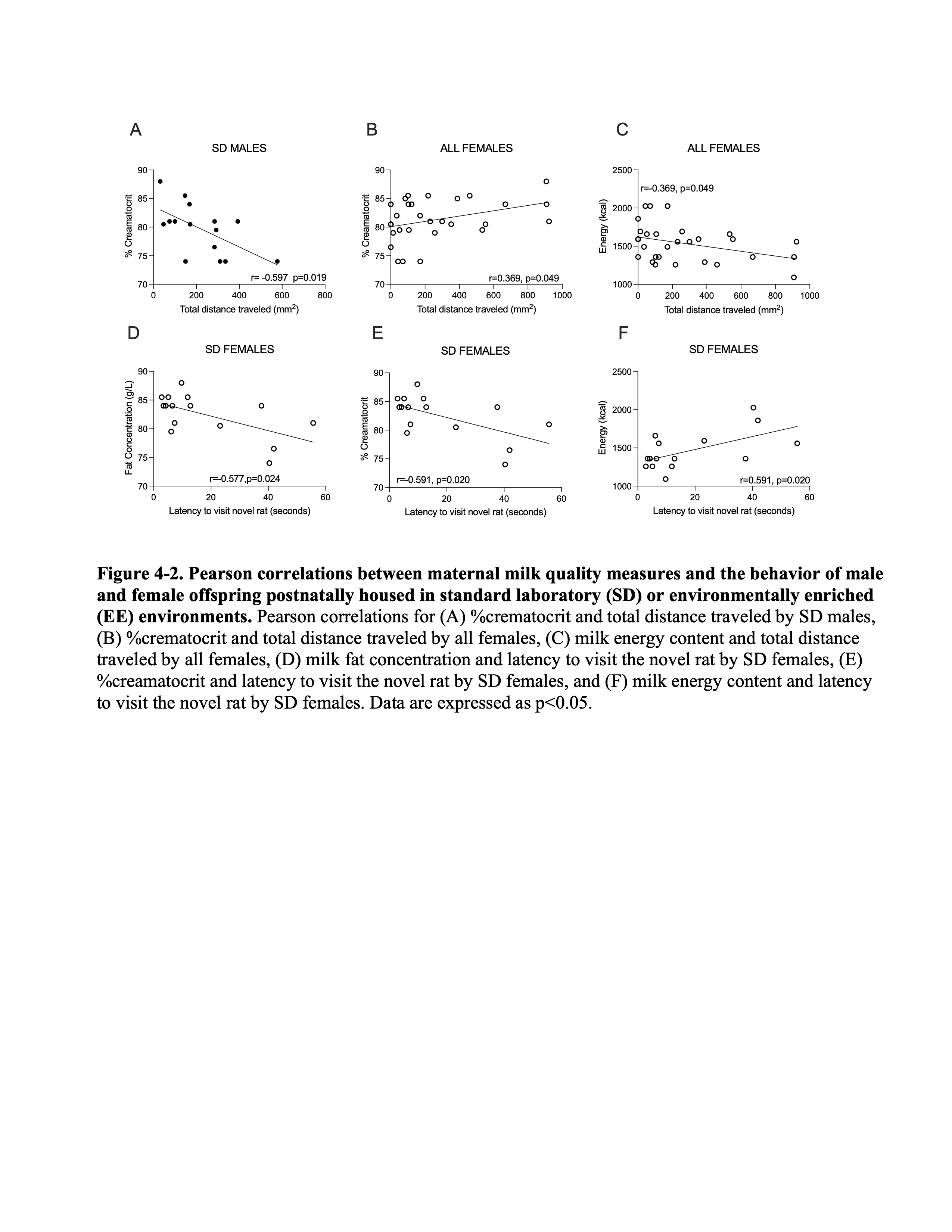

Supplement: Extended Data Figure 4-2 — Pearson correlations between maternal milk quality measures and the behavior of male and female offspring postnatally housed in standard laboratory (SD) or EE environments. Pearson correlations between distance traveled (mm2) and % creamatocrit for (A) postnatally housed SD males and (B) all females regardless of housing. C, Milk energy content (kcal/l) and total distance traveled by all females. Milk fat concentration (g/l; D), % creamatocrit (E), and milk energy content (kcal/l; F) correlations for latency to visit the novel rat by postnatally housed SD females. Data are expressed as p < 0.05. Download Figure 4-2, TIF file. [file enu-eN-NWR-0148-22-s07.tif]

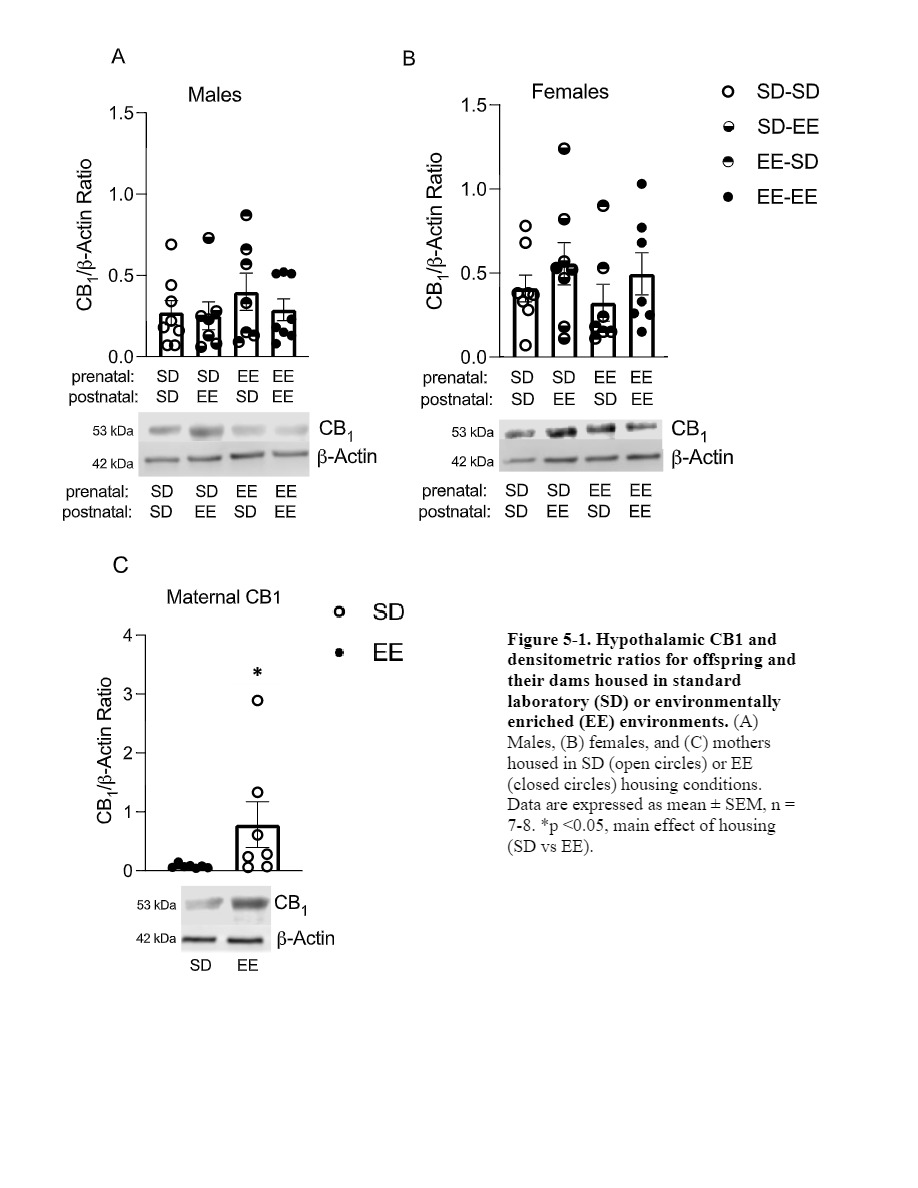

Supplement: Extended Data Figure 5-1 — Hypothalamic CB1 and densitometric ratios for offspring and their dams housed in standard laboratory (SD) or EE environments. Males (A), females (B), and mothers (C) housed in SD (open circles) or EE (closed circles) housing conditions. Data are expressed as mean ± SEM, n = 7–8; *p < 0.05, main effect of housing (SD vs EE). Download Figure 5-1, TIF file. [file enu-eN-NWR-0148-22-s08.tif]
